# Institutions and the Drug Supply

**Published:** 1915-04-10

**Authors:** 


### INSTITUTIONS AND THE DRUG SUPPLY.

It is common knowledge that since the war began the prices of a large number of commonly prescribed drugs have been steadily increasing, and those who read the notes on the drug market which appear regularly in The Hospital need hardly be reminded that almost each week has witnessed an advance in the value of some of these drugs. Early in the war there appeared to be some ground for the belief that there would be a limit to these advances, because there seemed to be promises that British manufacturers were about to commence an irresistible attack on German trade by producing in this country a goodly proportion of those medicinal products which had hitherto been procured almost exclusively from Germany. But up till the present we have waited in vain for any sign of this formidable campaign, and those who have delayed making their purchases in the hope that prices would be brought down to more reasonable levels have been disappointed. It is true that a few of the drugs for our supplies of which we were formerly dependent upon Germany are now being made in this country. For instance, salicylate of soda and acetyl-salicylic acid are being made here, but the extent of the output can be estimated from the fact that both these drugs are quoted at seven or eight times the pre-war value, and that it would be difficult to buy them in large quantities at any price. Indeed, it seems probable that unless further supplies are received from some neutral country stocks in this country will soon be nearly exhausted. The consumption of these products is so exceedingly large that British makers cannot be expected to supply the normal demand at short notice; and even if they could prices would necessarily be greatly above the normal because of the vastly increased cost of carbolic acid, which is wanted to make explosives for killing rather than medicines for healing. As to the many other drugs which our own manufacturers have failed to produce in the necessary commercial quantities, it would be unjust to attribute this failure to lack of enterprise or, indeed, to lack of knowledge.

It must be remembered that our manufacturing druggists have been obliged to increase enormously their output of galenical preparations and of the drugs they had been accustomed to produce. Never in the history of the world has there been such activity in the druggists' laboratories; in our own country the production of drugs to supply the demand caused by the series of epidemic diseases that has characterised an unusually wet and depressing winter would have sufficed to make the drug market busier than usual. But, in addition to this, our manufacturers have been called upon to supply drugs for thousands and thousands of our own sick and wounded soldiers, and to furnish medicines for the treatment of the Armies of our Allies. All this has had to be accomplished with a shortage of workmen, for the druggists have furnished their fair share of recruits to the new Armies. Under the circumstances, therefore, it is not surprising that our own manufacturing chemists are still unable to produce the drugs which Germany used to supply to the markets of the world.

During the first six or seven months of the war fair supplies of many of the wanted drugs reached British ports from the United States of America and other neutral countries, but such supplies will in future be seriously curtailed, if not cut off, as a result of the stoppage of Germany's export trade; for America will have no surplus supplies to send to us if she cannot replenish her stocks from the main source. It is therefore evident that no reduction in the prices of most of the drugs affected by the war can be expected. Indeed, it is probable that the values will continue to advance, and those institutions which still defer the purchase of necessary supplies could only justify their attitude if there were grounds for believing that an early termination of the war was probable. It is almost certain that no relief of the situation can be expected until the war is over. Such drugs as the salicylates, phenacetin, phenazone, barbitone, acetanilide, sulphonal, and, in fact, practically all the synthetic drugs, are getting scarcer and dearer. The prices of morphine, codeine, atropine, cocaine, and a long list of other important products will almost certainly be maintained, and will most probably advance. A method which some institutions are finding it convenient to adopt is that of giving contracts for the supply of drugs at prices according to an agreed current wholesale list, less a fixed rate of discount. The prices are, of course, subject to market fluctuations, since quotations sometimes vary from day to day. Under the circumstances this system is fair both to contractors and to institutions.

